# Demonstration of highly specific toxicity of the **α**-emitting radioimmunoconjugate^211^At-rituximab against non-Hodgkin's lymphoma cells

**DOI:** 10.1054/bjoc.2000.1453

**Published:** 2000-10-26

**Authors:** E Aurlien, R H Larsen, G Kvalheim, Ø S Bruland

**Affiliations:** Department of Oncology, The Norwegian Radium Hospital, Montebello, Oslo, N-0310; Department of Chemistry, University of Oslo, Section D, Box 1033 Blindern, Oslo, N-0315; Clinical Stem Cell Laboratories, The Norwegian Radium Hospital, Montebello, Oslo, N-0310, Norway

**Keywords:** clonogenic survival, lymphoma cells, bone marrow, α-emitter, radioimmunoconjugate, rituximab

## Abstract

The ability of an α-emitter conjugated to a chimaeric anti-CD20 monoclonal antibody to kill selectively human B-lymphoma cells in vitro is reported. Two B-lymphoma cell lines RAEL and K422, and normal haematopoietic progenitor cells from human bone marrow aspirates were incubated with^211^At-rituximab (Rituxan® or MabThera™) and plated in clonogenic assays for survival analyses. Following 1 h incubation with^211^At-rituximab, in concentrations which gave an initial activity of 50 kBq ml^–1^, a high tumour cell to normal bone marrow cell toxicity ratio was obtained; 4.1 to 1.0 log cell kill. Biodistribution studies of^211^At-rituximab in Balb/c mice showed similar stability as that of the iodinated analogue. The data indicate that testing of^211^At-rituximab in human patients is warranted. © 2000 Cancer Research Campaign


British Journal of Cancer (2000) **83**(10), 1375–1379
© 2000 Cancer Research Campaign
doi: 10.1054/ bjoc.2000.1453, available online at http://www.idealibrary.com on IDEAL®

### Demonstration of highly specific toxicity of the α-emitting radioimmunoconjugate $^{211}$At-rituximab against non-Hodgkin's lymphoma cells

**E Aurlien[1], RH Larsen[2], G Kvalheim[3] and ØS Bruland[1]**

[1]The Norwegian Radium Hospital, Department of Oncology, Montebello, N-0310 Oslo; [2]University of Oslo, Department of Chemistry, Section D, Box 1033 Blindern, N-0315 Oslo; [3]The Norwegian Radium Hospital, Clinical Stem Cell Laboratories, Montebello, N-0310 Oslo, Norway

**Summary** The ability of an α-emitter conjugated to a chimaeric anti-CD20 monoclonal antibody to kill selectively human B-lymphoma cells in vitro is reported. Two B-lymphoma cell lines RAEL and K422, and normal haematopoietic progenitor cells from human bone marrow aspirates were incubated with $^{211}$At-rituximab (Rituxan® or MabThera™) and plated in clonogenic assays for survival analyses. Following 1 h incubation with $^{211}$At-rituximab, in concentrations which gave an initial activity of 50 kBq ml$^{-1}$, a high tumour cell to normal bone marrow cell toxicity ratio was obtained; 4.1 to 1.0 log cell kill. Biodistribution studies of $^{211}$At-rituximab in Balb/c mice showed similar stability as that of the iodinated analogue. The data indicate that testing of $^{211}$At-rituximab in human patients is warranted. © 2000 Cancer Research Campaign

**Keywords**: clonogenic survival; lymphoma cells; bone marrow; α-emitter; radioimmunoconjugate; rituximab

Most patients with relapsed non-Hodgkin's lymphoma (NHL) are incurable by current conventional treatment. High-dose chemotherapy with stem cell support is effective only to patients with chemosensitive disease (Armitage, 1993). Hence, new treatment modalities are needed. Various immunotherapeutic approaches, including the use of native monoclonal antibodies (MoAbs), immunotoxins and radioimmunoconjugates, have been investigated (for review see Multani and Grossbard, 1998). MoAbs against the CD20 antigen given to patients with relapsed B-cell NHL yielded response rates of about 30–50% (McLaughlin et al, 1998). When conjugated to the β-emitting isotopes $^{131}$I (Press et al, 1995; Kaminski et al, 1996) or $^{90}$Y (Knox et al, 1996), responses in up to 86% were seen (Multani and Grossbard, 1998; Liu et al, 1998). Radioimmunotherapy is dose-limited mainly by haematological toxicity, unless stem cell support is given.

The α-emitter $^{211}$At has several favourable physical properties compared to the currently used β-emitters. Thus the half-life ($t_{1/2}$) of $^{211}$At is only 7.2 h, whereas $^{131}$I and $^{90}$Y have half-lives of 8.0 days and 64 h, respectively. Alpha particles from $^{211}$At have an average energy of 6.8 MeV and their range in soft tissue is only 55–80 μm, i.e. a few cell diameters. Because of the low range of the α-radiation in tissues, need for patient shielding is greatly reduced, allowing α-radioimmunotherapy to be given on an out-patient basis (Larsen et al, 1999). Moreover, the high LET (linear energy transfer) of $^{211}$At, about 97 keV μm$^{-1}$, is close to the optimum value for high RBE (relative biological effectiveness) (Brown, 1986). Alpha emitters are also generally associated with low oxygen enhancement ratio and virtual absence of dose–rate effects (Barendsen et al, 1965; Hall, 1994). The short range of the α-emitters implies that the radiation dose is delivered to the cell or within its close vicinity. This fact, together with the targeting ability of the MoAb, makes α-particle-emitting immunoconjugates attractive for radioimmunotherapy against dispersed cells and micrometastases.

The present study was performed to explore the ability of $^{211}$At-rituximab to selectively inactivate NHL tumour cells with acceptable bone marrow damage.

*Received 18 April 2000*
*Revised 13 July 2000*
*Accepted 18 July 2000*

*Correspondence to*: ØS Bruland

### MATERIALS AND METHODS

### Haematopoietic cells and lymphoma cell lines

Fresh mononuclear bone marrow (BM) cells from healthy volunteers were used. BM may contain various amounts of B-lymphocytes (CD20$^+$), from < 1% up to 15–20%. Peripheral blood progenitor cells (PBPC) were harvested in the recovery phase following chemotherapy and G-CSF (in patients with other diagnoses than NHL), and enriched CD34$^+$ haematopoietic cells (from PBPC) were used. Two B-cell NHL cell lines in exponential growth, RAEL (high-grade) and K422 (low-grade) (Dyer et al, 1990), expressing the CD20 antigen, were studied.

### Monoclonal antibody

A chimaeric anti-CD20 MoAb with murine variable regions and human $IgG_1$-kappa constant regions, IDEC-C2B8 or rituximab, was used. This MoAb is marketed by IDEC Pharmaceutical Corporation, San Diego CA and by Genentech Inc, San Francisco CA in the USA under the name of Rituxan®, and in Europe by Hoffmann-La Roche Ltd, Basel, Switzerland under the name MabThera™. The CD20 antigen is expressed at high density on malignant B-cells as well as normal B-lymphocytes, but not on early B-cells and stem cells. Within the time-frame of $^{211}$At decay, the CD20 antigen is virtually stable on the cell surfaces (Reff et al, 1994).

### Radionuclides and labelling

Astatine-211 was produced in a cyclotron at the Department of Physics, University of Oslo (Larsen et al, 1994 *a*) using the $^{209}Bi(\alpha, 2n)$ $^{211}At$ reaction. Commercially available $^{125}I$ (New England Nuclear, Billerica MA, USA) was used for the iodine labelling. Rituximab was radiolabelled with $^{211}At$ and $^{125}I$ and purified as previously described for other proteins (Larsen et al, 1994 *b*). The resulting radioimmunoconjugates, $^{211}At$-rituximab and $^{125}I$-rituximab, were sterile-filtered prior to the biological experiments, using a Millex-GV 0.22 μm filter (Millipore, Bedford MA, USA).

In the present experiments the specific activity varied from 10–75 MBq $mg^{-1}$. The specific binding fraction of $^{211}At$-labelled rituximab was determined in a one-point assay. Cell-bound radioimmunoconjugate was measured in triplicate after incubation at room temperature for 1–2 h, using approximately $10^7$ cells in 0.3 ml medium. An average of 36.4% of the labelled antibody (range 17.3–54.1%) bound to antigen-positive RAEL cells, whereas non-specific binding of $\leq 1.8\%$ was measured on the antigen-negative osteosarcoma cell line OHS. A binding fraction of 36% in the one-point assay corresponds to an immunoreactive fraction at infinite antigen excess (Lindmo et al, 1984) of about 50–60%, as verified experimentally (data not shown).

### Cell killing activity of $^{211}At$-rituximab

Different amounts of $^{211}At$-rituximab were added to a fixed concentration of $10^6$ cells $ml^{-1}$. Initial radioactivity concentrations (kBq $ml^{-1}$) in the test tubes were counted in a LKB Wallac 1277 Gammamaster gammacounter (Wallac Oy, Turku, Finland). Two experimental series with different incubation time were performed: A 1 h incubation series to study the efficacy of cell-bound radioimmunoconjugate; and a 4 h incubation series to simulate the prolonged circulation in vivo of IgG-type radioimmunoconjugates. Four hours are similar to the effective half-life of the radiopharmaceutical in the body, as indicated in mice experiments. After the incubation, under soft stirring for 1 or 4 h, the cells were washed in culture medium and centrifuged three times. Then survival was measured in clonogenic assays, and compared to that observed in the absence of radioactivity.

The number of clonogenic tumour cells remaining after exposure to $^{211}At$-rituximab was assessed by a modified version (Kvalheim et al, 1987) of the Courtenay and Mills soft agar clonogenic assay (Courtenay and Mills, 1978). For evaluation of toxicity on haematopoietic cells the CFU-c (colony forming unit cell) assay with METHOCULT H4433 medium (Stem Cell Technologies Inc, Vancouver, Canada) was used. The toxicity of the labelled MoAbs was also tested on more primitive (stem cell-near) haematopoietic cells, using the LTC-IC (long-term culture initiating cell) assay (Eaves et al, 1991). Briefly, the cells were seeded on a pre-irradiated (20 Gy) human bone marrow stroma mono-layer in the medium MYELOCULT H5100 (Stem Cell Technologies Inc), supplemented with 50 μM hydrocortisone, and cultured for 5 weeks before secondary culture in CFU-c assay.

### Therapeutic gain

The relationship between the initial concentration of radioactivity (kBq $ml^{-1}$) and clonogenic survival was, in the range tested, best fitted by quadratic regression. The concentration of $^{211}At$-rituximab giving a survival of 10% or 37% ($A_{10}$ or $A_{37}$) was calculated from the survival curves. A therapeutic gain factor (TGF) for tumour cells relative to haematopoietic cells exposed to $^{211}At$-rituximab, was calculated using the following equation:

$$TGF_{37} = A_{37,\ BM}/A_{37,tumour}$$

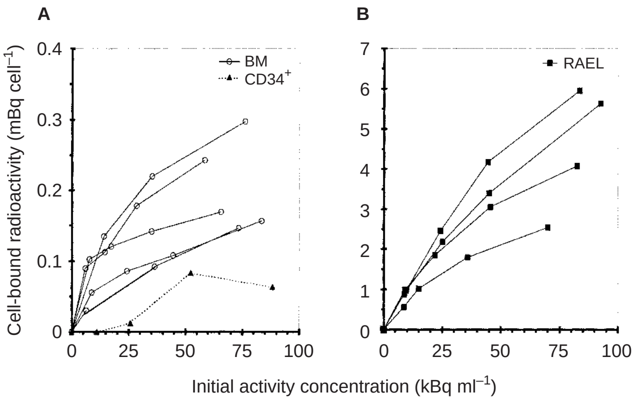

**Figure 1** Cell bound $^{211}At$-rituximab related to the initial activity concentration. Radioactivity (mBq $cell^{-1}$) bound to normal bone marrow cells (BM) and to $CD34^+$-selected haematopoietic cells (**A**) and to the lymphoma cell line RAEL (**B**). Note differences in scale on the y-axis

### Biodistribution

Biodistribution was studied in Balb/c mice. Astatine-211 and $^{125}I$ were examined in a 'paired label' fashion where 30 kBq of $^{125}I$-labelled and 120 kBq of $^{211}At$-labelled rituximab in 100 μl were co-injected into the tail vein of each animal. Approximately 4 μg rituximab were injected per mouse. At various time-points three mice were sacrificed, the organs were dissected out and weighed, the radioactivity contents were measured and the percentage of injected dose per g of tissue was determined.

### Statistical analyses and curve estimations

SPSS 8.0, EXCEL 97 for Windows, and SigmaPlot (Scientific Graphic Software, version 2, Jandel Corporation) were used in the statistical analyses, plots and graphs. The input values for the survival curves were calculated by the mean $\log_{10}$ anti-log of surviving fractions for separate doses in the individual experiments (presented in the Figures). Survival curves were fitted to the data using quadratic regression of $\log_{10}$ survival. The 95% confidence intervals (CI) of the survival curves were calculated using $\log_{10}$ of the 95% CI from each point on the survival curves.

### RESULTS

### Cell-bound radioactivity

Cell-bound radioactivity (mBq $cell^{-1}$), measured after 1 h incubation, is presented in Figure 1A for normal BM and $CD34^+$ cells and in Figure 1B for the RAEL cells. It is seen that, as expected, much more radioactivity was bound to the tumour cells than to the normal BM cells.

 © 2000 Cancer Research Campaign

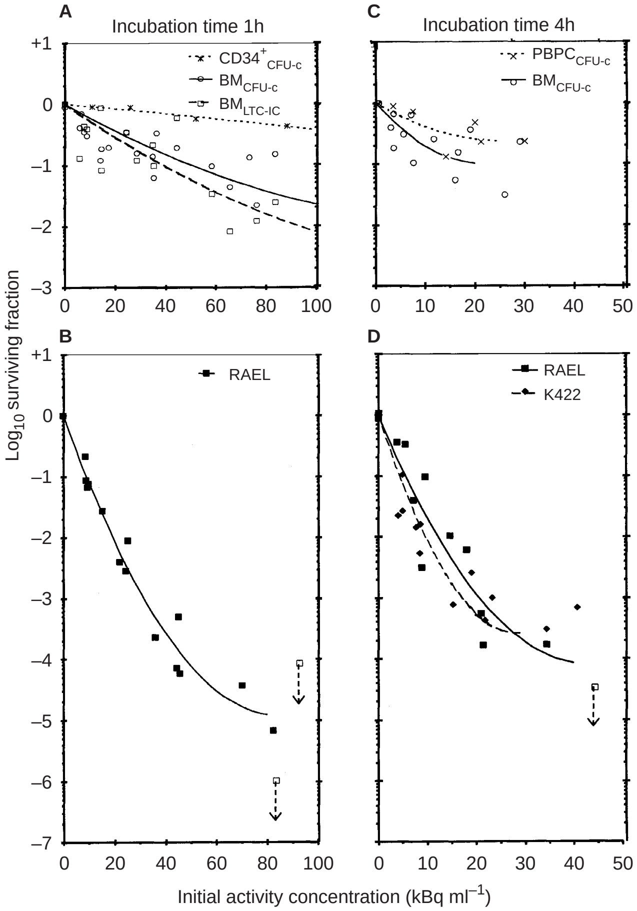

**Figure 2** Clonogenic survival of haematopoietic cells and lymphoma cells related to initial concentration of radioactivity (kBq ml$^{-1}$) of $^{211}$At-rituximab. Incubation times were 1 h (**A** and **B**) and 4 h (**C** and **D**). The logarithmic mean surviving fractions from each experiment (3–9 parallels at each point), and the quadratic regression lines are marked. The open symbols with arrows (**B** and **D**) represent points where no colonies were observed at the highest seeded cell concentration. (**A**) BM, evaluated in both CFU-c and LTC-IC assays, as well as CD34$^+$ cells plated in CFU-c assay, are compared, while in (**B**) the surviving fractions of RAEL are shown. (**C**) Survival of BM and PBPC following 4 h incubation and (**D**) the two tumour cell lines, RAEL and K422.

### Survival of lymphoma cells and haematopoietic cells following exposure to $^{211}$At-rituximab

Clonogenic survival vs initial activity concentration of $^{211}$At-rituximab is shown in Figure 2. The data in Figure 2A represent experiments with BM cells from five different donors tested in CFU-c, four of them also examined in LTC-IC assays. In addition, one experiment with enriched CD34$^+$ cells is included in the figure. In Figure 2B four individual experiments with RAEL cells are shown. Comparison of Figure 2A and 2B (1 h measurements) demonstrates a highly significant, favourable survival of the BM cells compared to the malignant RAEL cells. From the survival curves the following mean $A_{10}$ values were estimated: 50.0 kBq ml$^{-1}$ for $BM_{CFU\text{-}c}$, 39.1 for $BM_{LTC\text{-}IC}$, 203.5 for $CD34^+_{CFU\text{-}c}$ and 9.0 for RAEL. An initial activity concentration of 50 kBq ml$^{-1}$ yielded corresponding log cell kill values of 1.00, 1.24, 0.20 and 4.13. In Table 1 the values are given with 95% CI.

BM cells tested in LTC-IC were separated from cells in standard CFU-c assay just before plating. Hence, these stem cells were exposed to the same crossfire irradiation from B-cells in the pellets before seeding. The experiments showed a tendency of higher toxicity on BM evaluated in LTC-IC as compared to CFU-c assay, but the differences were not significant, $P = 0.36$ (paired samples t-test, two-tailed). In contrast, the toxicity towards CD34$^+$ cells was lower for the tested initial concentrations of activity (Figure 2A).

In Figure 2C and 2D the results of 4 h measurements are shown. Since 4 h incubation may reflect a more realistic time schedule with regard to the effective half-life of $^{211}$At-rituximab administered to patients, the study was extended with these series. The data in Figure 2C and 2D represent three individual experiments for each tumour cell line, BM from three different donors and two PBPC products. Again the $^{211}$At-rituximab was found to be far more toxic to the tumour cells than to the haematopoietic cells. However, neither between BM and PBPC nor between the two tumour cell lines were significant differences found. Calculations from the survival curve parameters give the following mean $A_{10}$ values (kBq ml$^{-1}$): 22.8 for BM, 5.6 for RAEL and 4.3 for K422. Such low survival level was not observed for PBPC in the given range of initial activity concentrations. At an initial activity concentration of 25 kBq ml$^{-1}$, the mean log cell kills were 1.01 for $BM_{CFU\text{-}c}$, 0.61 for $PBPC_{CFU\text{-}c}$, 3.39 and 3.54 for RAEL and K422, respectively (Table 1).

The amounts of radioactivity added in this study correspond to a rituximab concentration of less than 8 µg ml$^{-1}$. In a control experiment with unlabelled rituximab at similar concentrations as used with the radioimmunoconjugate, no significant effect on survival, neither of the RAEL nor the BM cells, was observed (data not shown). This indicates that the radioisotope was essential for the effectiveness of $^{211}$At-rituximab in the present experiments. The concentrations used represent less than 3.5% of the serum concentration obtained in clinical trails using therapeutic doses of native rituximab (McLaughlin et al, 1998).

**Table 1** $A_{10}$ values[a] and log cell kills[a] following exposure to $^{211}$At-rituximab

| Cells | $BM_{CFU\text{-}c}$ | $BM_{LTC\text{-}IC}$ | $CD34^+_{CFU\text{-}c}$ | $PBPC_{CFU\text{-}c}$ | RAEL | K422 |
|---|---|---|---|---|---|---|
| Incubation time – 1 h | | | | | | |
| $A_{10}$ (kBq ml$^{-1}$) | 50.0 | 39.1 | 203.5 | – | 9.0 | – |
| | (45.4–56.4) | (27.9, 45.4) | (167.1, –) | | (7.7, 9.8) | |
| Log cell kill | 1.00 | 1.24 | 0.20 | – | 4.13 | – |
| at 50 kBq ml$^{-1}$[b] | (0.91–1.10) | (1.09, 1.61) | (0.12, 0.28) | | (3.95–4.50) | |
| Incubation time – 4 h | | | | | | |
| $A_{10}$ (kBq ml$^{-1}$) | 22.8 | – | – | – | 5.6 | 4.3 |
| | (12.6, –) | | | | (5.5–6.0) | (4.1–4.6) |
| Log cell kill | 1.01 | – | – | 0.61 | 3.39 | 3.54 |
| at 25 kBq ml$^{-1}$[b] | (0.86–1.14) | | | (0.44–0.71) | (3.18–3.96) | (3.38–3.70) |

[a]Mean (95%CI); [b]initial activity concentration

© 2000 Cancer Research Campaign

**Table 2** Therapeutic gain factor at 37% cell survival ($TGF_{37}$)[a] following exposure to $^{211}$At-rituximab

| | RAEL to | | | | K422 to | |
|---|---|---|---|---|---|---|
| Incubation time | $BM_{CFU-c}$ | $BM_{LTC-IC}$ | $CD34^+_{CFU-c}$ | $PBPC_{CFU-c}$ | $BM_{CFU-c}$ | $PBPC_{CFU-c}$ |
| 1 h | 5.2 ± 0.7 | 4.2 ± 1.2 | 26.8 ± 3.9 | – | – | – |
| 4 h | 2.3 ± 0.5 | – | – | 5.0 ± 2.8 | 3.0 ± 0.7 | 6.5 ± 3.7 |

[a]Mean ± SD

**Table 3** Biodistribution[a] of $^{211}$At- and $^{125}$I-rituximab in mice

| | 1 h | | 3.5 h | | 10.75 h | | 23.5 h | |
|---|---|---|---|---|---|---|---|---|
| Tissue | $^{211}$At-rituximab | $^{125}$I-rituximab | $^{211}$At-rituximab | $^{125}$I-rituximab | $^{211}$At-rituximab | $^{125}$I-rituximab | $^{211}$At-rituximab | $^{125}$I-rituximab |
| Blood | 36.2 ± 2.9 | 37.9 ± 2.1 | 32.9 ± 1.9 | 32.3 ± 2.0 | 27.1 ± 1.2 | 28.5 ± 0.6 | 17.4 ± 1.4 | 20.1 ± 2.0 |
| Lung | 15.5 ± 3.6 | 15.7 ± 3.8 | 13.4 ± 1.5 | 12.6 ± 2.0 | 11.6 ± 1.3 | 11.1 ± 1.2 | 8.5 ± 1.9 | 8.7 ± 1.8 |
| Liver | 11.5 ± 1.5 | 13.0 ± 1.7 | 9.5 ± 1.0 | 10.5 ± 0.8 | 6.7 ± 0.7 | 8.2 ± 1.2 | 4.4 ± 1.2 | 4.2 ± 3.4 |
| Heart | 10.5 ± 0.8 | 10.1 ± 0.8 | 10.2 ± 0.9 | 11.2 ± 1.2 | 8.9 ± 1.3 | 8.4 ± 0.5 | 6.8 ± 0.9 | 6.9 ± 0.9 |
| Spleen | 8.1 ± 2.7 | 8.7 ± 1.9 | 7.9 ± 0.9 | 7.6 ± 0.7 | 7.3 ± 0.6 | 7.2 ± 0.2 | 4.6 ± 0.8 | 5.0 ± 0.6 |
| Kidney | 7.9 ± 0.8 | 9.1 ± 0.8 | 8.5 ± 1.1 | 9.0 ± 1.2 | 6.5 ± 1.3 | 7.7 ± 0.9 | 4.1 ± 0.1 | 5.2 ± 0.6 |
| Muscle | 1.0 ± 0.4 | 1.2 ± 0.3 | 1.6 ± 0.1 | 1.6 ± 0.1 | 1.6 ± 0.1 | 1.8 ± 0.1 | 1.5 ± 0.3 | 1.8 ± 0.3 |
| Bone | 2.8 ± 0.7 | 2.9 ± 0.3 | 2.8 ± 0.5 | 2.9 ± 0.1 | 3.0 ± 0.2 | 3.2 ± 0.2 | 2.0 ± 0.4 | 2.9 ± 0.2 |

[a]Values expressed as % of i.v. injected dose of $^{211}$At- and $^{125}$I-rituximab per gram tissue, mean ± SD

### Therapeutic gain

Both 1 h and 4 h incubation with $^{211}$At-rituximab gave favourable therapeutic windows between tumour cells and haematopoietic cells. The average $TGF_{37}$ ± SD, is presented in Table 2. In the 1 h series $TGF_{37}$ between RAEL and $BM_{CFU-c}$ was 5.2 in average. $TGF_{37}$ for tumour cells vs $BM_{CFU-c}$ in the 4 h series were 2.3 for RAEL and 3.0 for K422.

### Biodistribution in mice

Table 3 presents the tissue distribution of $^{211}$At-rituximab and $^{125}$I-rituximab in eight organs of the mice, within a time-frame of 1–23.5 h. The biodistribution of $^{211}$At-rituximab was similar compared to that of $^{125}$I-rituximab, and revealed the general characteristics of IgG radiolabelled MoAbs, i.e. high initial activity levels in blood and blood-rich tissues.

### DISCUSSION

The reported experiments were designed to assess targeted α-emitter exposure to dispersed B-lymphoma cells and haematopoietic progenitor cells.

The normal bone marrow cells generally showed a low sensitivity following exposure to $^{211}$At-rituximab. BM contains various amounts of B-lymphocytes, ranging from < 1% up to 20%, which may explain the considerable variation in cell-bound activity observed in different experiments. The difference in toxicity on the BM cells, reflected in the survival curve (Figures 2A and 2C), could possibly be explained by crossfire irradiation of the antigen-negative clonogenic cells from B-cells in the pellets before seeding. This is further supported by the very low toxicity observed with $CD34^+$-enriched cells, which had low binding of $^{211}$At-rituximab, due to the virtual absence of B-cells in this cell population. Furthermore, PBPC products, which contain about 0.5–1% B-cells, also showed a trend towards lower cytotoxicity from the radioimmunoconjugate compared to BM, although the difference was not statistically significant. Neither was the survival significantly different between BM cells tested concurrently in the CFU-c and the LTC-IC assay.

Astatine-211-labelled rituximab was highly cytotoxic to both high- and low-grade NHL cells when measured after 4 h incubation, and no significant difference in survival between the two cell lines was found (Figure 2D).

In our experiments, $A_{37}$ following 1 h incubation was 3.8 and 19.5 kBq $ml^{-1}$ for RAEL and $BM_{CFU-c}$, respectively, whereas the calculated value for $CD34^+$ cells was as high as 100.9 kBq $ml^{-1}$. Hence, $A_{37}$ for $CD34^+$ cells was almost identical to the values previously reported for human cell lines exposed to non-specific $^{211}$At-BSA (bovine serum albumin) (Larsen et al, 1994*a*).

The biodistribution profile indicated that the quality of the immunoconjugate had not been significantly reduced by labelling with $^{211}$At and also that the stability of the conjugate in vivo was adequate. As with $^{131}$l-labeled compounds a blocking agent should be used for therapeutic purposes to minimize radiation dose to the thyroid for $^{211}$At (Larsen et al, 1998).

The current study confirms previous reports indicating that α-emitting immunoconjugates may have selective anti-tumour effect. One study by Harrison and Royle (1987) used $^{211}$At conjugated to a T-cell antibody (OX7) to treat a T-cell lymphoma in mice and increased the median survival time of mice and probably 'cured' more than half of the animals (Harrison and Royle, 1987). Later Behr and colleagues (1999) compared the therapeutic efficacy and toxicity of the α-emitter $^{213}$Bi with that of the β-emitter $^{90}$Y, linked to a monovalent Fab′ fragment, in a human colonic cancer xenograft model in nude mice (Behr et al, 1999). They concluded that radioimmunotherapy with α-emitters may be therapeutically more effective than conventional β-emitters. Recently pharmacokinetics and dosimetry of a $^{213}$Bi-labelled anti-CD33-antibody in patients with leukaemia was reported (Sgouros et al, 1999).

Clinical studies with radioimmunoconjugates ($^{131}$I and $^{90}$Y) in lymphomas have mainly involved murine MoAbs. Using a chimeric MoAb like rituximab in radioimmunotherapy could

 © 2000 Cancer Research Campaign strongly reduce HAMA (human anti-mouse antibody) responses in patients. However, the prolonged biological half-life of the humanized MoAbs could cause problems with increased radiation dose to normal tissues, and hence a decreased therapeutic index, when isotopes with longer half-life (days) are utilized (Bruland, 1995; Multani and Grossbard, 1999). The use of a relatively short-lived isotope like $^{211}$At will make those differences in biological half-life less relevant.

As pointed out, $^{211}$At also has several advantages from a radiation-protection point of view. More than 99% of its radiation energy is from α-particles. The short half-life (7.2 h) could reduce the hospitalization time, and the short tissue-range of the α-particles (maximum 80 μm) would simplify the protection of the staff and environment (Larsen et al, 1999).

A BM progenitor cell survival of 5% should be sufficient for recovery without stem-cell support. This survival level of $BM_{CFU\text{-}c}$ was observed at an initial activity concentration of 70.3 kBq ml$^{-1}$ following 1 h incubation. The corresponding log cell kill of RAEL was as high as 4.8. Consequently there may be a significant therapeutic window if non-myeloablative doses of $^{211}$At-rituximab are explored clinically.

In conclusion, our study shows that rituximab labelled with $^{211}$At exerts selective cytotoxicity to NHL tumour cells in vitro. The effect of the radioimmunoconjugate will now be investigated in patients with relapsed B-cell lymphomas.

### ACKNOWLEDGEMENTS

The work was financially supported by The Norwegian Cancer Society, grant no. 97014 and 96070. Rituximab (MabThera™) was a gift from Roche Norway A/S. Thanks are due to Dr LS Rusten, Clinical Stem Cell Laboratories, the Norwegian Radium Hospital, Oslo, Norway, for instruction in the LTC-IC method, and to Mr E Olsen, Department of Physics, University of Oslo for performing cyclotron irradiations to produce $^{211}$At. We are indebted to Professor A Pihl for constructive criticism of the manuscript.

### REFERENCES

Armitage JO (1993) Treatment of non-Hodgkin's lymphoma. *N Engl J Med* **328**: 1023–1030

Barendsen GW, Koot CJ, Van Kersen GR, Bewley DK, Field SB and Parnell CJ (1965) The effect of oxygen on impairment of the proliferative capacity of human cells in culture by ionizing radiations of different LET. *Int J Radiat Oncol Biol Phys* **10**: 317–327

Behr TM, Behe M, Stabin MG, Wehrmann E, Apostolidis C, Molinet R, Strutz F, Fayyazi A, Wieland E, Gratz S, Koch L, Goldenberg DM and Becker W (1999) High-linear energy transfer (LET) alpha versus low-LET beta emitters in radioimmunotherapy of solid tumors: therapeutic efficacy and dose-limiting toxicity of 213Bi-versus 90Y-labeled CO17-1A Fab′ fragments in a human colonic cancer model. *Cancer Res* **59**: 2635–2643

Brown I (1986) Astatine-211: its possible applications in cancer therapy. *Int J Radiat Appl Instrum A* **37**: 789–798

Bruland OS (1995) Cancer therapy with radiolabelled antibodies. An overview. *Acta Oncol* **34**: 1085–1094

Courtenay VD and Mills J (1978) An *in vitro* colony assay for human tumours grown in immune-suppressed mice and treated *in vivo* with cytotoxic agents. *Br J Cancer* **37**: 261–268

Dyer MJ, Fischer P, Nacheva E, Labastide W and Karpas A (1990) A new human B-cell non-Hodgkin's lymphoma cell line (Karpas 422) exhibiting both t(14;18) and t(4;11) chromosomal translocations. *Blood* **75**: 709–714

Eaves CJ, Cashman, JD and Eaves AC (1991) Methodology of long-term culture of human hemopioetic cells. *J Tiss Cult Meth* **13**: 55–62

Hall EJ (1994) Linear energy transfer and relative biological effectiveness. In: *Radiobiology for the Radiologist*, 4th edn, pp 153–164. Philadelphia: JB Lippincott Company

Harrison A and Royle L (1987) Efficacy of astatine-211-labeled monoclonal antibody in treatment of murine T-cell lymphoma. *National Cancer Institute Monogr*: 157–158

Kaminski MS, Zasadny KR, Francis IR, Fenner MC, Ross CW, Milik AW, Estes J, Tuck M, Regan D, Fisher S, Glenn SD and Wahl RL (1996) Iodine-131-anti-B1 radioimmunotherapy for B-cell lymphoma. *J Clin Oncol* **14**: 1974–1981

Knox SJ, Goris ML, Trisler K, Negrin R, Davis T, Liles TM, Grillo LA, Chinn P, Varns C, Ning SC, Fowler S, Deb N, Becker M, Marquez C and Levy R (1996) Yttrium-90-labeled anti-CD20 monoclonal antibody therapy of recurrent B-cell lymphoma. *Clin Cancer Res* **2**: 457–470

Kvalheim G, Fodstad O, Pihl A, Nustad K, Pharo A, Ugelstad J and Funderud S (1987) Elimination of B-lymphoma cells from human bone marrow: model experiments using monodisperse magnetic particles coated with primary monoclonal antibodies. *Cancer Res* **47**: 846–851

Larsen RH, Bruland OS, Hoff P, Alstad J, Lindmo T and Rofstad EK (1994*a*) Inactivation of human osteosarcoma cells in vitro by 211 At-TP-3 monoclonal antibody: comparison with astatine-211-labeled bovine serum albumin, free astatine-211 and external-beam X rays. *Radiat Res* **139**: 178–184

Larsen RH, Hoff P, Alstad J and Bruland OS (1994*b*) Preparation and quality control of $^{211}$At-labelled and $^{125}$I-labelled monoclonal antibodies. Biodistribution in mice carrying human osteosarcoma xenografts. *Journal of Labelled Compounds and Radiopharmaceuticals* **XXXIV**: 774–785

Larsen RH, Slade S and Zalutsky MR (1998) Blocking [211At]astatide accumulation in normal tissues: preliminary evaluation of seven potential compounds. *Nucl Med Biol* **25**: 351–357

Larsen RH, Murud KM, Akabani G, Hoff P, Bruland ØS and Zalutsky MR (1999) $^{211}$At- and $^{131}$I-labelled bisphosphonates with high in vivo stability and bone accumulation. *J Nucl Med* **40**: 1197–1203

Lindmo T, Boven E, Cuttitta F, Fedorko J and Bunn PA Jr. (1984) Determination of the immunoreactive fraction of radiolabeled monoclonal antibodies by linear extrapolation to binding at infinite antigen excess. *J Immunol Methods* **72**: 77–89

Liu SY, Eary JF, Petersdorf SH, Martin PJ, Maloney DG, Appelbaum FR, Matthews DC, Bush SA, Durack LD, Fisher DR, Gooley TA, Bernstein ID and Press OW (1998) Follow-up of relapsed B-cell lymphoma patients treated with iodine-131-labeled anti-CD20 antibody and autologous stem-cell rescue. *J Clin Oncol* **16**: 3270–3278

McLaughlin P, Grillo-López AJ, Link BK, Levy R, Czuczman MS, Williams ME, Heyman MR, Bence-Brukler I, White CA, Cabanillas F, Jain V, Ho AD, Lister J, Wey K, Shen D and Dallaire BK (1998) Rituximab chimeric anti-CD20 monoclonal antibody therapy for relapsed indolent lymphoma: half of patients respond to a four-dose treatment program. *J Clin Oncol* **16**: 2825–2833

Multani PS and Grossbard ML (1998) Monoclonal antibody-based therapies for hematologic malignancies. *J Clin Oncol* **16**: 3691–3710

Multani PS and Grossbard ML (1999) Antibody-based therapy for lymphoma. *Adv Oncol* **15**: 23–29

Press OW, Eary JF, Appelbaum FR, Martin PJ, Nelp WB, Glenn S, Fisher DR, Porter B, Matthews DC and Gooley T (1995) Phase II trial of 131I-B1 (anti-CD20) antibody therapy with autologous stem cell transplantation for relapsed B cell lymphomas. *Lancet* **346**: 336–340

Reff ME, Carner K, Chambers KS, Chinn PC, Leonard JE, Raab R, Newman RA, Hanna N and Anderson DR (1994) Depletion of B cells in vivo by a chimeric mouse human monoclonal antibody to CD20. *Blood* **83**: 435–445

Sgouros G, Ballangrud AM, Jurcic JG, McDevitt MR, Humm JL, Erdi YE, Mehta BM, Finn RD, Larson SM and Scheinberg DA (1999) Pharmacokinetics and dosimetry of an alpha-particle emitter labeled antibody: 213Bi-HuM195 (anti-CD33) in patients with leukemia. *J Nucl Med* **40**: 1935–1946

*© 2000 Cancer Research Campaign*
